# Cutaneous Leishmaniasis Hampers COVID-19: A Controlled Cross-Sectional Study in High-Burden Endemic Areas of Iran

**DOI:** 10.1007/s44197-023-00179-0

**Published:** 2024-01-08

**Authors:** Mehdi Bamorovat, Iraj Sharifi, Mehdi Shafiei Bafti, Setareh Agha Kuchak Afshari, Mohammad Reza Aflatoonian, Ali Karamoozian, Abdollah Jafarzadeh, Raheleh Amirzadeh, Ahmad Khosravi, Zahra Babaei, Farzane Safa, Fatemeh Sharifi, Amireh Heshmatkhah

**Affiliations:** 1 Leishmaniasis Research Center, Kerman University of Medical Sciences, Kerman, Iran; 2 Institute for Studies in Medicine History, Persian and Complementary Medicine, Iran University of Medical Sciences, Tehran, Iran; 3 Deputy for Health, Kerman University of Medical Sciences, Kerman, Iran; 4 Medical Mycology and Bacteriology Research Center, Kerman University of Medical Sciences, Kerman, Iran; 5 Research Center for Modeling in Health, Institute for Futures Studies in Health, Kerman University of Medical Sciences, Kerman, Iran; 6 Department of Immunology, Kerman University of Medical Sciences, Kerman, Iran; 7 Research Center for Social Determinants of Health, Institute for Futures Studies in Health, Kerman University of Medical Sciences, Kerman, Iran; 8 Research Center for Tropical and Infectious Diseases, Kerman University of Medical Sciences, Kerman, Iran; 9 Dadbin Health Clinic, Kerman University of Medical Sciences, Kerman, Iran

**Keywords:** Cutaneous leishmaniasis, COVID-19, Severity, Trained innate immunity, Cross-protection

## Abstract

**Introduction:**

Emerging infectious diseases such as SARS-CoV-2 can cause pandemics and create a critical risk for humans. In a previous pilot study, we reported that the immunological responses induced by cutaneous leishmaniasis (CL) could decrease the incidence and severity of COVID-19. In this large-scale case–control study, we assessed the possible relationship between mortality and morbidity of COVID-19 in healed CL persons suffering scars compared to cases without CL history.

**Methods:**

This controlled cross-sectional study was conducted between July 2020 and December 2022 in the endemic and high-burden areas of CL in southeastern Iran. In the study, 1400 previous CL cases with scars and 1,521,329 subjects who had no previous CL were analyzed. We used R 4.0.2 to analyze the data. Firth’s bias reduction approach corresponding to the penalization of likelihood logistic regression by Jeffreys was also employed to influence the variables in the dataset. Also, a Bayesian ordinal logistic regression model was performed to explore the COVID-19 severity in both case and referent groups.

**Results:**

The occurrence and severity rate of COVID-19 in CL scar cases are significantly less than in the non-CL control group, while in the CL scar subjects, patients with critical conditions and mortality were not observed. The morbidity (OR = 0.11, CI 0.06–0.20 and *P* < 0.001) and severity of COVID-19 in previous cases with CL scars were significantly diminished than that in the control group (credible interval − 2.57, − 1.62).

**Conclusions:**

The results represented a durable negative relationship between cured CL and COVID-19 incidence and severity. Additional studies seem necessary and should be designed to further validate the true impact and underlying mechanistic action of CL on COVID-19.

**Graphical abstract:**

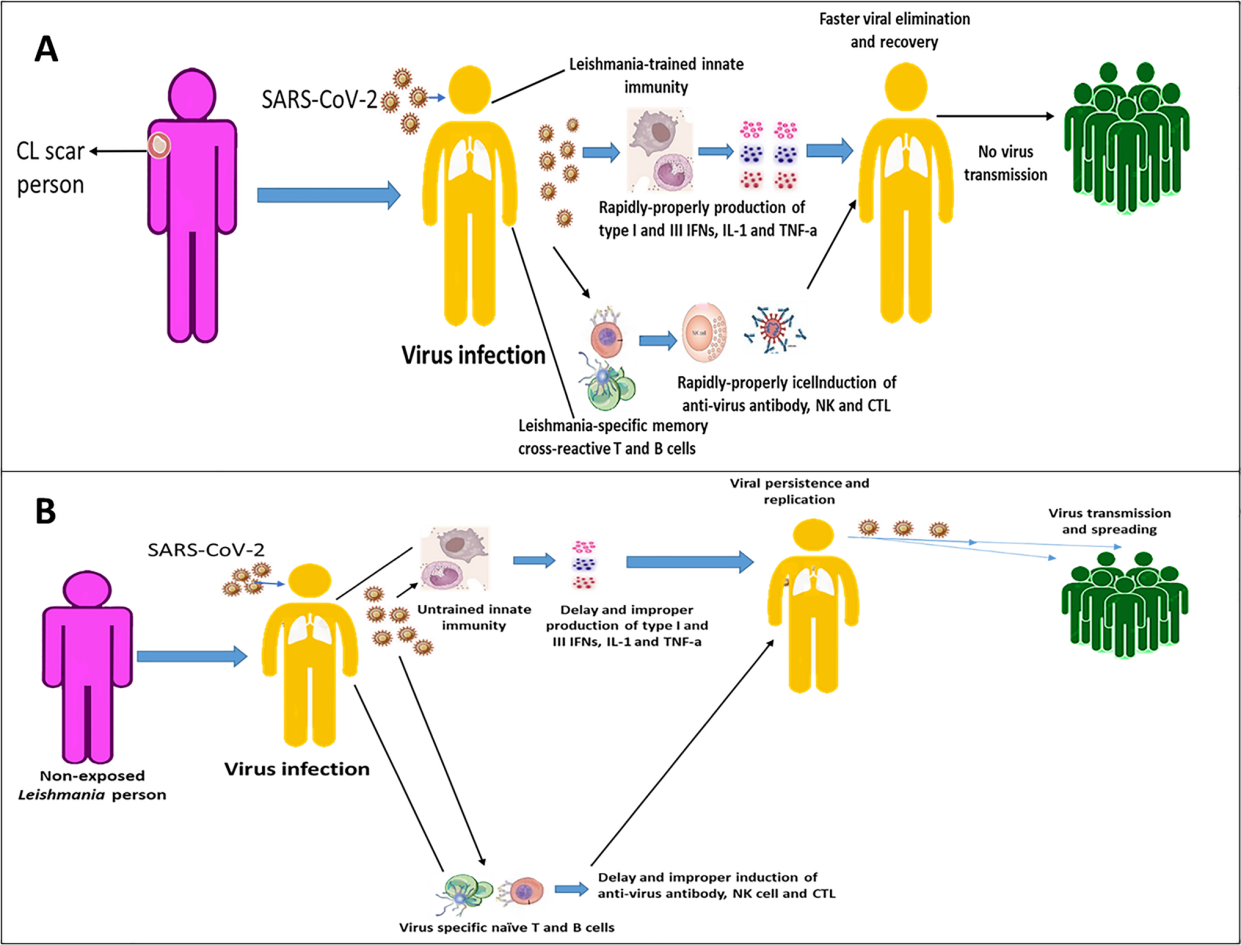

## Introduction

Leishmaniasis as a neglected and tropical disease has increased and expanded in several geographical areas, diverse hosts, and vectors [1]. For human infection, nearly 23 *Leishmania* species have been reported [1]. Different forms of the disease are endemic in over 100 nations [1–3]. Cutaneous leishmaniasis (CL) is the most prevalent type due to *Leishmania tropica* (anthroponotic CL; urban form) and *Leishmania major* (zoonotic CL; rural form). The Global Burden of Disease study expected CL morbidity to be 4.6 million cases in 2019 [3].

Anthroponotic CL (ACL) and zoonotic CL (ZCL) are endemic in several parts of Iran such as Kerman province [4]. In Kerman, CL is generally due to *L*. *tropica* which is transmitted from human to human by the *Phlebotomus (Ph*.*) sergenti* [5]. According to the current studies performed in the Kerman and Bam districts, all examined CL cases were infected by *L*. *tropica* species [6–9]. However, in Baft and Arzuiyeh County, CL cases usually were infected with *L. major* [10]*.*

In leishmaniasis, both innate and adaptive immune responses are indistinguishably associated together as the cytokines generated by cells of the innate system control the result and extent of the adaptive immunity [11]. Therefore, the evoked immune responses are necessary to be firmly regulated to evade immune-mediated damage in host tissue. In a study by Ajdary et al., assessing T-cell responses to *Leishmania* species antigen in an experimental model, Th2 cell response was central in active CL patients, and Th1 was considered the group of cured patients [12]. The clearance and control of *L. major* development involved principally cytotoxic CD8+ T lymphocytes and CD4+ helper T lymphocytes and the production of IFN-g [13]. Non-responsive skin lesions in mice are linked with a few TCD4+ [14]. A report indicated that local immune limits, such as cellular and cytokines liberated in the lesion, control organisms and protect against the pathological process [11].

The effector T helper (Th) 1 phenotype associated with cell-mediated immune response performs a more pivotal role in the protection [15]. Interleukin-12 (IL-12) and gamma interferon (IFN-γ) play a critical role in defense against *Leishmania* infection via the induction of the polarization of Th1 cells. Th1 pathways, particularly IFN-γ, and tumor necrotizing factor (TNF-α), activate macrophages leading to *Leishmania* eradication through the production of proteolytic enzymes, nitric oxide (NO), and various oxygen radical species [15]. Differentiation of the Th2 subset is led by IL-4 and increasing susceptibility via inhibition of macrophages and IL-12 production [15]. Development of leishmaniasis is associated with predominant Th2- and Treg cell-relevant responses and high-level production of TGF-β, IL-10, IL-4, and IL-5 [16].

On the other hand, the current emergence of severe respiratory syndrome due to coronavirus was a global health problem with critical concerns. A varied range of mechanisms like immune imbalances, hyper-inflammatory responses, lymphopenia, enormous reproduction of viral particles, cytokine storm, coagulopathy, and wide cell death resulting in COVID-19 pathologic processes, leading to multiple-organ collapse, particularly severe respiratory set of symptoms [17].

The primary barrier of resistance against viral infections is innate interferons (IFNs), as the sensible production of appropriate quantities of IFNs are capable of restricting viral infections [18]. Proper local IFNcross-1 and 2 responses in the initial phases of coronavirus infection can eliminate COVID-19 or restrict its proliferation, preventing the development of the disease to moderate and severe stages [18]. Nevertheless, if the primary IFN reactions are ineffective in controlling COVID-19, the virus proliferates in the pulmonary system, through blood flow leading to different organs and causing vast tissue destruction [18]. In the adaptive immune response, effector Th1 phenotypes, particularly IL-2 and IFN-γ, stimulate natural killer cells (NK) and cytotoxic CD8^+^ T lymphocytes (CTLs) to diminish viral infection by eliminating infected cells [19]. IFN-γ enhances parasite control by triggering infected macrophages to generate leishmanicidal products such as nitric oxide prone to killing intramastigote parasites, likewise one of the critical cytokines for the protection against viral infections. However, the initial IFN-γ responses are vital to controlling coronavirus; if it ceases to function, the virus actively replicates and further leads to immense tissue destruction. During SARS-CoV-2 replication and in many patient investigations, IFN-γ-producing T lymphocyte quantities were higher [20].

The findings from some studies declared that one of the issues that can reduce COVID-19 severity is coexisting infection with microbial and parasitic diseases [21]. In a pilot case–control field assessment, Bamorovat and colleagues explained how CL can reduce the morbidity and mortality of COVID-19 [22]. A cohort observation proposed that coinfection with intestinal parasites induced a milder COVID-19 [23]. Also, other studies claimed that several parasitic infections can confer protection against COVID-19 and other pulmonary disorders [24, 25]. The cross-protection due to CL may lead to lower incidence and severity of COVID-19 would meaningfully benefit endemic areas in developing nations, particularly the Mediterranean Basin. Certainly, a smaller rate of COVID-19 cases and casualty rates throughout the pandemic have been reported from these regions [3].

In CL infection, the elimination of *Leishmania* is largely carried out by CD8^+^ and CD4^+^ helper T lymphocytes, in particular by the presentation of IFN-γ [26]. In addition to exerting potent anti-viral activity, IFN-γ enhances parasite control by stimulating macrophages to produce anti-leishmania products, such as NO and ROS. In children, COVID-19 is mainly mild, as it produces great levels of IFN-γ limiting virus spreading [27].

In a previous preliminary study, we reported that the immunological responses due to CL could reduce the burden of SARS-CoV-2 infection [22]. In this large-scale study, we evaluated the possible relationship between mortality and morbidity of COVID-19 in healed CL persons who had previous scars compared to those with no history of CL during the 2 years of the COVID-19 pandemic in a well-defined CL endemic area in southeastern Iran.

## Methods

### Ethics Statement

This study was granted ethical agreement (Ethics No. IR.KMU.REC.1400.513, commitment No. 400000704) by the Kerman University of Medical Sciences.

Volunteer participants with confirmed cured CL scar were considered the CL scar group and those without symptoms of CL scar were regarded as the control group. Various interviews and face-to-face meetings were determined and conducted with the local health officials and participants to explain the goal, process, and probable gains. The cases and guardians completely were informed about the possible benefits and small risks in the context of the investigation. During the meeting, the assessors guaranteed that the queries were well-informed by the patients or their guardians. In this study, cured cases with CL participated freely. For individuals or caretakers, written informed consent was obtained. They were allowed to decline the study for any reason at any stage. All recorded data from all cases were kept confidential and they were managed free of charge during the course of study. During the clinical assessments, subjects who were suspicious of having probable comorbidities were referred to higher tertiary hospitals for more follow-up examination.

### Study Site and Design

This controlled cross-sectional study was performed between July 2020 to December 2022 in the endemic and high-burden areas of urban CL caused by *L. tropica* in Kerman province. The province is located in the southeast of the country. The province consists of different types of climatic settings providing suitable conditions for the propagation of CL [28]. The average yearly rainfall is below 150 mm. The study was carried out at the Health Centers of several counties of Kerman province, the main referral clinics for CL control actions. The Health Centers are linked to the Leishmaniasis Research Center and the School of Medicine in Kerman. These Health Centers are accountable for managing CL cases that have been referred from several zones within the province. There was a case report form (CRF) for each CL person that recorded demographic and clinical status. All previously registered CL cases have been diagnosed by conventional tests including direct microscopic examination, culture media, and/or molecular techniques [28].

### Participants

#### CL Scar Group

**Fig. 1:**
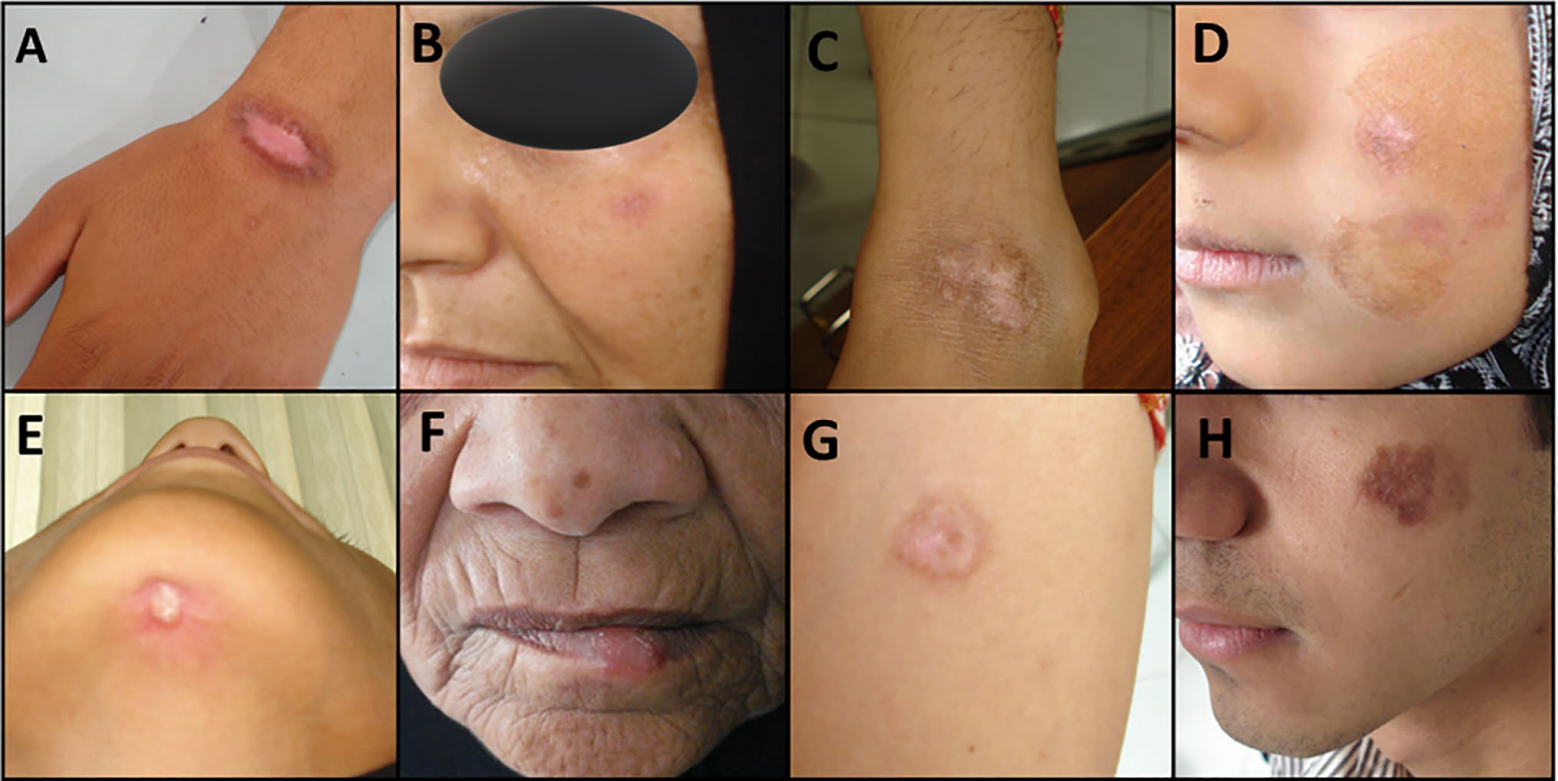
Representative images of previously healed skin lesions (scars) taken from patients with different anatomical locations of cutaneous leishmaniasis cases under study areas within the Kerman province (**A** and **G**: Hand; **B**, **D,** and **H**: Face; **C**: Leg; **F**: Lip; **E**: Under the chin)

This study was performed as a census, although about 52% (1400 from 2700) of overall previous CL cases with scars were included. This was mainly due to the vast and remote areas under study and the inaccessibility of the subjects, migration, displacements, and/or possible death. In this study, all healed CL patients had a history of scars and were diagnosed by direct smear (stained with Giemsa) or culture media and molecular examination [28]. Suspected CL persons were identified by the direct microscopic smear preparation at the referral. Direct smears were achieved from the CL lesions and then dried, methanol fixed, stained with Giemsa, and finally, detected by microscope for the finding of the parasite (amastigote; Leishman body). CL-healed cases were evaluated prospectively for SARS-CoV-2 infection. This group included individuals who had CL scars and prospectively followed up for COVID-19 contraction. Figure 1 shows the scar lesions (after being cured) of CL cases.

#### Control Group

In suspected individuals with SARS-CoV-2 who were referred to the educational hospitals affiliated with the Kerman University of Medical Sciences, the SARS-CoV-2 infection was confirmed and recognized using the Multiple One-Step quantitative real-time PCR technique by the commercial COVITECH kit [22]. In the control group (persons without a CL history who were infected with COVID-19), the data were taken in cooperation with the Statistics Center of the university. The control group included all the confirmed SARS-CoV-2-infected cases referring to all the Medical Centers. Similar endemic communities within the counties made both case and control groups. The groups were matched involving age, sex, and socioeconomic features. Hence, they were similarly exposed to the infection with SARS-CoV-2.

### Statistical Analyses

We used R 4.0.2 to analyze the data. Firth’s bias reduction approach corresponding to the penalization of likelihood logistic regression by Jeffreys was also employed to influence the variables in the dataset. In this model, there was no essential concern about accurate coefficient estimation regarding a few incident data in the studied dataset. Also, the p-value, odds ratio (OR), and the regression coefficient were calculated.

Besides, to analyze the severity (mortality) of SARS-CoV-2 in both groups, Bayesian inference in ordinal logistic regression was applied. It is necessary to mention that standard statistical methods to analyze this dataset might have inappropriate and incorrect consequences (because the occurrence of the events remains moderately low at some degrees). As with the prior distribution, normal distribution was applied. Last, in R software, the weights and logistic packages were applied for analysis.

## Results

### Findings of the CL Scar Group

In general, in the CL scar group, 1400 laboratory-established patients with previous CL scar (including 685 males and 615 females) were assessed and analyzed for probable infection with COVID-19. In the CL scar group, from 12 persons with confirmed COVID-19, 7 cases (58.3%) exhibited a mild form of COVID-19. Overall, 5 persons (41.7%) were hospitalized, and neither of them showed critical COVID-19 symptoms nor died (Table 1).

**Table 1:** The incidence and severity of COVID-19 in cutaneous leishmaniasis (CL)-cured cases and the control group during the two-year pandemic period based on gender and age

The severity of COVID-19	Incidence (no.)	Gender (no.)		Age group (year) (no.)			
		Male	Female	1–15	15–30	30–50	> 50
Patients with CL							
Mild	7	4	3	0	0	3	4
Hospitalized	5	3	2	0	2	0	3
Critical	0	0	0	0	0	0	0
Death	0	0	0	0	0	0	0
Total	12	7	5	0	2	3	7
Patients without CL							
Mild	93,567	47,719	45,848	4678	13,099	29,941	45,849
Hospitalized	20,228	10,234	9994	957	2937	6406	9928
Critical	1011	516	495	51	142	323	495
Death	2379	1312	1067	30	87	368	1894
Total	117,185	59,781	57,404	5716	16,265	37,038	58,166

### Findings of the Control Group

The control group was selected from the general population covered by the services of the Kerman University of Medical Sciences (1521,329 healthy individuals without CL scars). Overall, in the control group, 117,185 subjects (7.70%) with contracted laboratory-confirmed COVID-19 infection were assessed for COVID-19 outcomes.

**Fig. 2:**
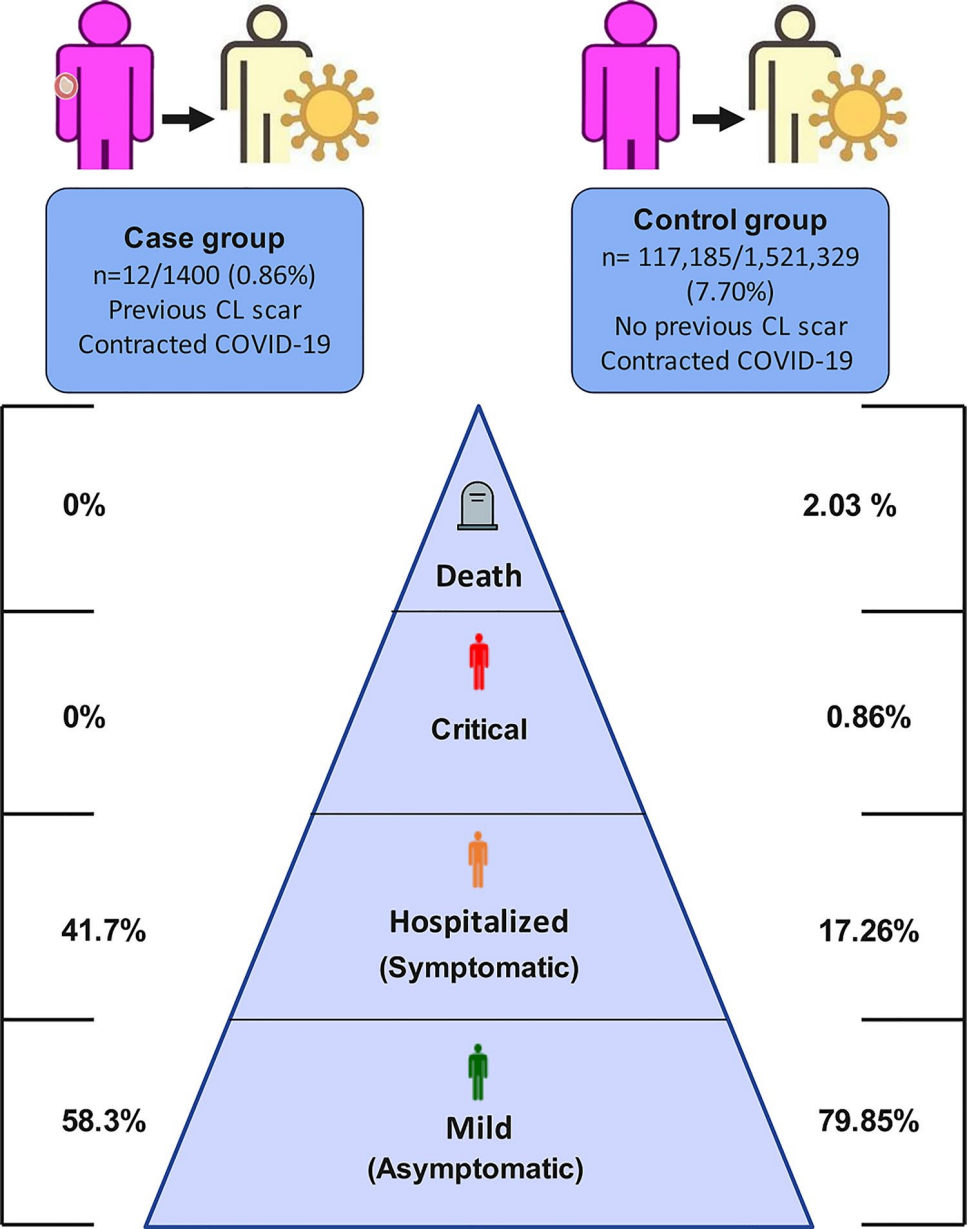
The severity rate of COVID-19 based on clinical symptoms in exposed and non-exposed *Leishmania* (case and control) groups

In control group, from 117,185 persons with confirmed COVID-19, 93,567 cases (79.85%) exhibited the mild forms of COVID-19, 20,228 cases (17.26%) were hospitalized, 1011 cases (0.86%) displayed critical symptoms and 2,379 (2.03%) were dead from COVID-19-related complications (Fig. 2) (Table 1).

### Analytical Findings

The results showed that there was a durable negative relationship between CL-healed cases and COVID-19 incidence and severity. According to Firth’s Bias-Reduced logistic regression, the burden of the SARS-CoV-2 in CL-healed cases was considerably declined in terms of incidence (OR = 0.11, CI 0.06–0.20 and *P* < 0.001) and severity (credible interval: − 2.57, − 1.62) compared to that in the non-CL control group (Table 2).

**Table 2:** Statistical analyses of COVID-19 morbidity and mortality in the cutaneous leishmaniasis (CL)-cured cases compared to the non-CL control group

Variable	COVID-19		β	SE	OR	95% CI for OR	*p* value
	No	Yes					
Intercept			− 2.56	0.003			< 0.001
Patients without CL	1,521,329	117,185	0		1		
Patients with CL	1400	12	− 2.15	0.28	0.11	0.06–0.20	< 0.001

Variable	COVID-19					Mean	Std. dev	Credible interval	
	Total	Mild	Hospitalized	Critical	Death				
Patients with CL	1400	7	5	0	0	− 2.05	0.24	− 2.57	− 1.62
Patients without CL	1,521,329	93,567	20,228	1011	2379				

β coefficient regression, *SE* standard error, *OR* odds ratio, *CI* confidence interval

The credible interval (− 2.57, − 1.62) does not include zero and also considers the negativeness of the estimated coefficient as well as the lower and upper bounds of the probable interval. The findings confirm that the severity of COVID-19 in CL-cured cases is significantly less than that in the non-CL control group (Table 2).

## Discussion

Nowadays we breathe in a time where the growth of population, globalization, climate change, and emerging infectious diseases, can endanger public health and our socioeconomic condition. As a Coronaviridae family, SARS-CoV-2 is a highly transmissible coronavirus that emerged in December 2019 in locals of Wuhan town, China [29]. Emerging infectious diseases such as SARS-CoV-2 can produce pandemics and create a serious risk for which we are not ready. For this reason, prophylactic or therapeutic measures are vitally essential to limit or control the rate of morbidity and mortality instigated by the virus [30].

This study displayed those individuals with past CL scars had a considerably diminished COVID-19-related incidence and mortality relative to the negative CL scar group. The previous scar cases who recovered from CL had been infected between 2013 and 2020 (before the appearance of the COVID-19 pandemic). Clinical evidence indicates that the disease severity of affected individuals with SARS-CoV-2 was significantly minor in cases with the CL scar group than that in the non-CL subjects. The exact reasons for the greater resistance of persons with previously cured CL to SARS-CoV-2 infection remain to be elucidated in upcoming investigations. The induction of trained immunity, as well as memory cross-reactive lymphocytes, can confirm more resistance against COVID-19 in persons with previously cured CL. The immune system can be classified as innate immunity, which defends in a prompt and non-specific procedure, and acquired immune system, which is sluggish to function but can stimulate precise and strong responses by interactions among antigen-presenting cells (APCs) components, like T cells and B cells, and dendritic cells (DCs) [30, 31].

In host innate immunity, various cytokines (in particular IFNs) and natural killer (NK) cells act as the first line against viral infections. Considering COVID-19, a proper, timely, and efficient IFN response during the early phase of infection (especially the incubation period) can limit SARS-CoV-2 replication and prevent its spread. *Leishmania* parasite can have capabilities to amplify innate defense through a process called trained innate immunity. Trained immunity has been characterized as a nonspecific natural immune system capable of protecting humans against emerging infectious diseases [30, 31]. It can be postulated that a previous *Leishmania* infection could induce a rapid and proper pro-inflammatory cytokine response (such as type I/type III IFNs, IL-1β, TNF-a, and IL-6) in monocytes and macrophages when exposed to SARS-CoV-2. During the primary stage of SARS-CoV-2 infection, this rapid and proper cytokine response can effectively and quickly eliminate SARS-CoV-2, thereby reducing the disease’s severity and preventing virus transmission and spread within the community. Similarly, it has been reported that vaccination with BCG can confirm protection against various viral infections such as human papillomavirus, herpes simplex, and respiratory syncytial viruses through induction of the trained innate immunity [32, 33]. BCG vaccine produced against *Mycobacterium tuberculosis* infection can induce innate immune memory [34]. Experimentally, it was indicated that immunization with BCG can reduce viral load and lung damage in influenza A virus-infected mice [35]. According to several preliminary studies, it was reported that countries without universal BCG vaccination may be more susceptible to COVID-19 in comparison to neighboring countries performing general BCG vaccination programs [36]. Many current findings display a negative correlation between the incidence and mortality of COVID-19 and BCG vaccination [37]. Contact with several microbial infections during a life history could reinforce BCG-induced trained immunity, similar to a re-vaccination [38]. Hence, it is likely that innate immune memory caused by BCG vaccination at birth may confer protection against SARS-CoV-2. Therefore, BCG vaccination can persuade a trained immunity that can trigger a more effective immune response in infection with SARS-CoV-2.

In addition to non-specific *Leishmania*-trained innate immunity, *Leishmania*-specific memory cross-reactive T and B cells can contribute to the induction of effective adaptive immunity against SARS-CoV-2. Following leishmaniasis resolution, the generation of short-lived and long-lived memory T cells was indicated, which can mediate a faster and stronger secondary response [39]. Although the epitope mapping comparison of the *Leishmania* parasite and SARS-CoV-2 will determine the possible presence of some common T and B lymphocyte epitopes, it can be assumed that some immune-dominant T and B lymphocyte epitopes may be shared between these two pathogens. If the existence of the cross-reacting T and B cells is proved, they can contribute to the induction of specific immune responses to SARS-CoV-2 through the production of cytokines like IFN‐γ (recruiting NK cells and CTLs) and the generation of neutralizing anti-virus antibodies, respectively.

Innate lymphoid cells (ILCs) are innate cells that express vital roles in immune defense against microbial infections, modulation of adaptive immunity, substance remodeling, and homeostasis of hematopoietic and nonhematopoietic cells [40]. ILCs prepare immunological memory cells; this is shown that the innate immune memory cells are more complicated than formerly known. Using the ability of trained immunity for COVID-19 disease first needs an awareness of the path accountable for intermediating the immune cells and also training pathogens to motivate these immunological alterations [41].

On the contrary, there are varying ranges of interactions between pathogens and parasitic infections. One of the usual occurrences in nature is concomitant infections and is commonly related to parasites. There are many reports of this interplay ensuing in predisposition or confrontation among parasites, bacteria, and viral infections that have been explained [42]. Many effects of parasites on the immune structure are well demonstrated in concomitant infections, such as immunopotentiation and immunosuppression [42]. A study showed that b-glucan develops host immunity against *L. braziliensis* by the induction of trained immunity, especially IL-1 signaling and IL-32 g as significant mediators for the eradication of *Leishmania* ([43].

**Fig. 3:**
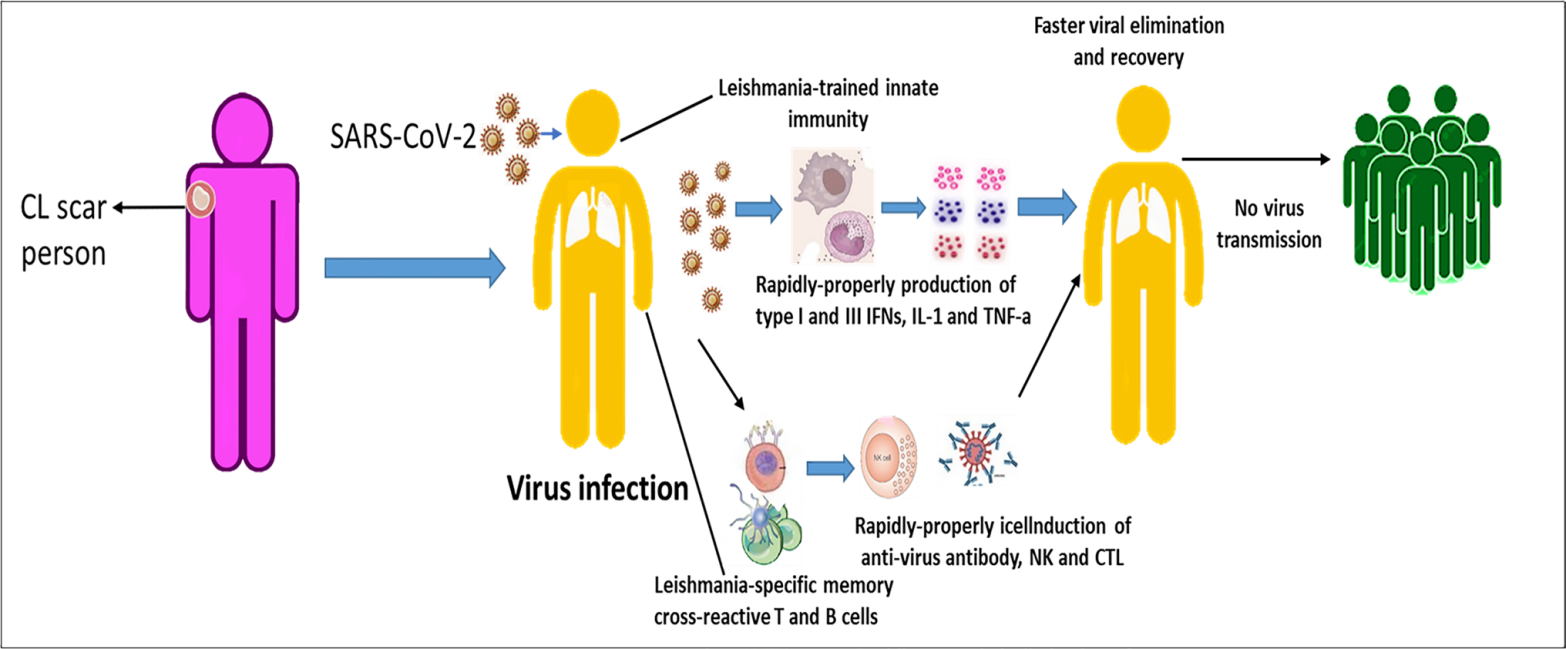
Possible mechanisms contributing to the resistance of *Leishmania*-infected persons against SARS-COV-2 infection

Figure 3 shows the presence of *Leishmania*-trained innate immunity and *Leishmania*-specific memory cross-reactive T and B cells contribute to the induction of rapid anti-virus immune responses eliminating the infection.

**Fig. 4:**
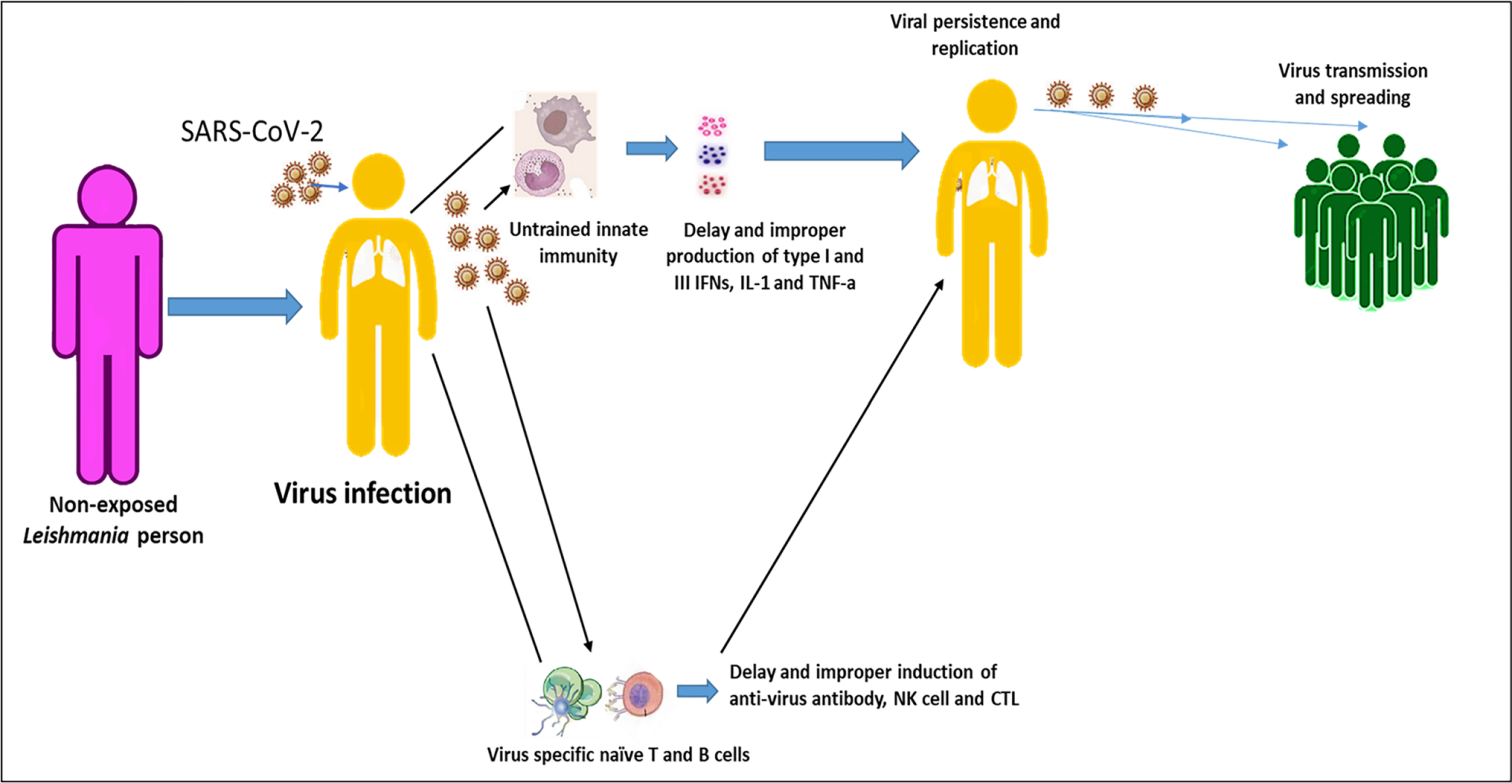
Susceptibility of the non-exposed *Leishmania*-infected person against SARS-COV-2 infection

Figure 4 shows the absence of *Leishmania*-trained innate immunity and *Leishmania*-specific memory cross-reactive T and B cells contribute to the delayed induction of anti-virus immune responses allowing infection persistence and spreading.

Chronic parasitic diseases can affect the host’s immune responses and change the clinical appearances of other infections, especially viruses [44]. One of the factors that affect the severity/mortality of SARS-CoV-2 is the activation of the immune response [45]. Lack of comorbid parasitic infection may raise the risk of COVID-19 severity [46]. Chronic infections with parasites are related to macrophage activation, ILC2, and T helper-2 (Th2). Such activities are convoyed by encouraging cytokines such as ILs 4,5 and 13 and developing responses of eosinophils, alongside IgE [45]. Throughout parasitic propagation, Th2 immune elements are attended by the stimulation action of regulatory T cells (Tregs) that are very significant in preserving parasitic persistence, and this function affects the immune response to other infectious diseases [47]. This is while severe SARS-CoV-2 is connected with expanding hyperinflammation [45]. Accordingly, Treg and Th2 responses for persistent parasite control in chronic parasite infections unbalanced Th1 responses that are found in severe COVID-19 and possibly modify the immune response of the reservoir host [48]. Many studies demonstrated that parasitic infections can defend against COVID-19 and many other respiratory syndromes [25, 49, 50]. A study recognized potential shared targets for immunity to SARS-CoV-2 by immune determinants’ shared common characteristics with *Plasmodium falciparum*. Probable cross-reactivity is proposed via human leukocyte antigen and consequent CD8+ T cell stimulation. The apparent immunodominant epitope maintenance between SARS-CoV-2 and *P. falciparum* thrombospondin-linked unidentified protein might inspire the low COVID-19 occurrence in the malaria-endemic areas by preparing immunity against virus replication in those formerly infected with *Plasmodium* [50]. Helminth parasites have coevolved outstanding approaches to interact with host immune responses, to restrict direct and bystander tissue injury through educating our immune elements to endure infection. In particular, immune responses are deeply predisposed by helminths transient through the lung (*hookworm, Ascaris lumbricoides,* and *Schistosoma mansoni*) or through their liberation of immunomodulators. Importantly, these effective immunomodulators can impact pulmonary immunity with adult worms lodging at distal locations. Helminths are perhaps a source of new treatments for pulmonary inflammatory diseases and essential research on helminth modulation of lung immune response will remain to bring new understanding into the processes that regulate pulmonary mechanisms in health and disease [49]. In another report, it was confirmed that helminth parasites including *Fasciola hepatica* can regulate host mechanisms connected to viral infection. Subsequently, it could be assumed that helminth parasites and their byproducts might affect SARS-CoV-2 entrance into host tissues and employ an anti-inflammatory consequence in COVID-19 patients [25].

Exposure to parasitic diseases on SARS-CoV-2, particularly those associated with helminth infection, may reduce the incidence and mortality of COVID-19 [48]. It seems that the elimination trend of parasitic diseases has caused some emerging infectious diseases to increase in recent years. However, a report has stated that parasitic infections intensify the risk of COVID-19 susceptibility [51]. Nonetheless, additional studies should be accomplished to determine the impact of parasitic coinfection on the reduction in incidence and severity in SARS-CoV-2 cases. Perhaps the induction of trained immunity in humans by *Leishmania* or other appropriate agents can protect against emerging infectious diseases such as COVID-19 or other emerging viruses in the future. Precise clinical studies appear necessary and should be designed to further validate this hypothesis. Moreover, possibly parasitic diseases that date back to the evolutionary times of life, even though they are considered pathogenic, have protected their hosts from emerging diseases and severe infections. It is believed that they have served a substantial role in the evolution and preservation of species and hindered some degenerative diseases and allergic reactions [52].

One of the study limitations is that Kerman is a vast province with numerous rural zones located in remote areas. All healed CL registered cases in this area were selected and included in the study and compared with the total population of the area. Because the CL cases are identified based on passive case detection surveillance, some cases might not be referred to, and their names were not recorded in the registry system. Due to the different nature and control strategy of the patients in both diseases; retroactive detection of CL scars, and patients with SARS-CoV2 (who were refereed and identified in the hospitals) the two groups were not comparable. Another possible limitation of the study was that the cases with CL might prevent social gatherings and as a result, they might avoid COVID-19 infection, although the social stigma in previously healed cases could be negligible. Conversely, the important strength of this study is the well-resourced Health surveillance system associated with the Kerman University of Medical Sciences and Leishmaniasis Research Center, its robust administrative office, and strong infrastructures that achieve the cases vigorously with experienced staff and expert physicians. We have exclusive ACL foci linked with our Research Center. Furthermore, this study is unique because no similar study has previously been conducted with this large-scale sample size.

## Conclusion

The findings confirm that the burden of COVID-19 disease in CL scar cases is significantly less than in the non-CL control group. In the CL scar group, patients with critical conditions and mortality were not observed. Perhaps there is a cross-protective effect against SARS-CoV-2 infection. Additional studies seem necessary and should be designed to further validate the true impact and underlying mechanistic action of CL on COVID-19.

## Data Availability

Data will be made available on reasonable request.

## Abbreviations

SARS-CoV-2: Severe acute respiratory syndrome 2
CL: Cutaneous leishmaniasis
COVID-19: Coronavirus disease 2019
OR: Odds ratio
Th: T helper
IFN-γ: Gamma interferon
TNF-α: Tumor necrotizing factor
NO: Nitric oxide
IL: Interleukin
TGF-β: Transforming growth factor beta
NK: Natural killer cells
CTLs: CD8^+^ T lymphocytes
CRF: Case report form
APCs: Antigen-presenting cells
BCG: Bacillus Calmette–Guerin
ILCs: Innate lymphoid cells
Tregs: Regulatory T cells
